# 2696. Impact of an Ultrasensitive *Cytomegalovirus* (CMV) Quantitative Nucleic Acid Test (qNAT) on CMV Detection and Therapy in Renal Transplant Recipients

**DOI:** 10.1093/ofid/ofad500.2307

**Published:** 2023-11-27

**Authors:** Vivek Beechar, Varun Phadke, Stephanie M Pouch, Christian P Larsen, Michael H Woodworth

**Affiliations:** Emory University School of Medicine, Atlanta, Georgia; Emory University, Atlanta, GA; Emory University School of Medicine, Atlanta, Georgia; Emory University School of Medicine, Atlanta, Georgia; Emory University, Atlanta, GA

## Abstract

**Background:**

Cytomegalovirus (CMV) infection in renal transplant recipients (RTR) impacts morbidity, mortality, and graft survival. Patients are risk-stratified based on their CMV serostatus as low risk (D-/R-), moderate risk (R+), and high risk (D+/R-). Most transplant centers conduct surveillance for CMV reactivation using quantitative nucleic acid testing (qNAT) at regular intervals for a fixed duration after transplantation. Antiviral treatment decisions are often guided by quantitative DNAemia. We hypothesize that the change in the qNAT platform to a newer, more sensitive assay lead to earlier CMV detection, longer antiviral treatment durations, and no difference in one-year all-cause mortality in moderate and high CMV risk RTR.

**Methods:**

We conducted a cohort study comparing RTRs monitored with the historical higher lower limit of quantification (LLOQ) qNAT (quantifies viral loads > 300 IU/mL) to the newer lower LLOQ qNAT assay (quantifies viral loads > 35 IU/mL). Patients were stratified by CMV serostatus for direct comparisons among moderate and high CMV risk patients. CMV viral load monitoring occurred at least monthly post-transplantation. Primary outcomes were antiviral treatment duration and time to detection of CMV DNAemia.

**Results:**

Both the moderate and high CMV risk groups tested with the lower LLOQ qNAT assay exhibited lower peak viral loads and lower first detected viral loads (Table 1). Comparing moderate risk groups, lower LLOQ patients experienced longer CMV DNAemia and corresponding antiviral treatment durations. All-cause mortality risks were not significantly different between the lower LLOQ and higher LLOQ moderate risk patients. In Kaplan Meier analyses, moderate risk patients tested using the lower LLOQ qNAT assay had CMV reactivation detected earlier following cessation of antiviral prophylaxis compared with patients tested using the higher LLOQ assay.

Table 1

**Figure:**
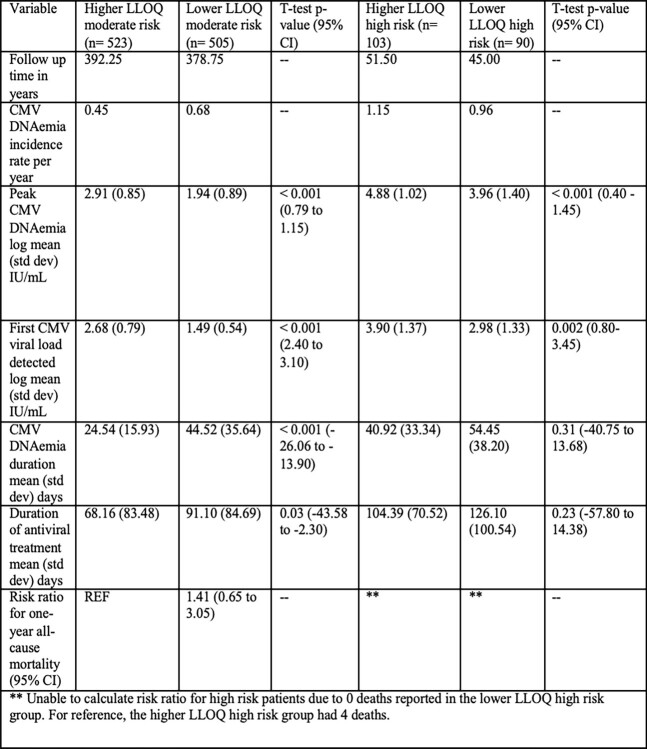

Lower LLOQ and higher LLOQ moderate and high-risk group comparisons for follow up time, CMV DNAemia incidence rate, peak CMV DNAemia, first CMV viral load detected, CMV DNAemia duration, and duration of antiviral treatment. Means, standard deviations, and t-tests are reported where applicable.

Moderate Risk KM Comparison

**Figure:**
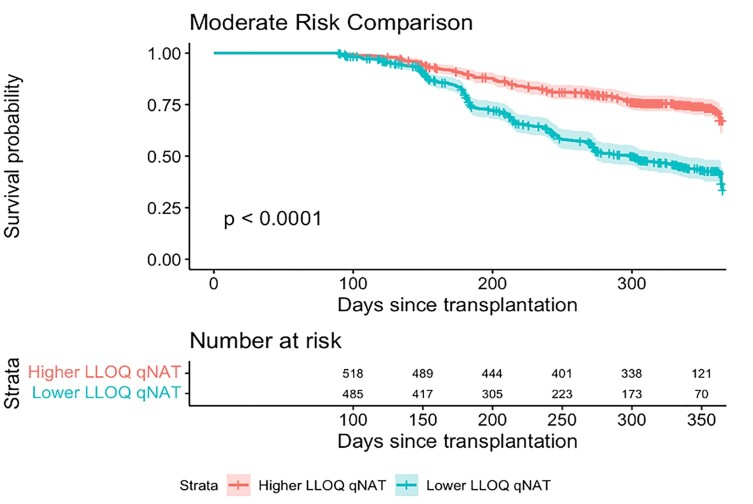

Kaplan Meier curve analysis comparing time to first episode of CMV DNAemia for higher LLOQ moderate risk patients (n=523) with lower LLOQ moderate risk patients (n=505) over a one year follow up period after transplantation. A log-rank test was performed and shows a statistically significant difference (p-value <0.0001) in time to CMV DNAemia between the two groups. The median survival for the lower LLOQ group was 300 days and could not be calculated for the higher LLOQ group given that the survival probability was greater than 50% at the end of the study period.

High Risk KM Comparison

**Figure:**
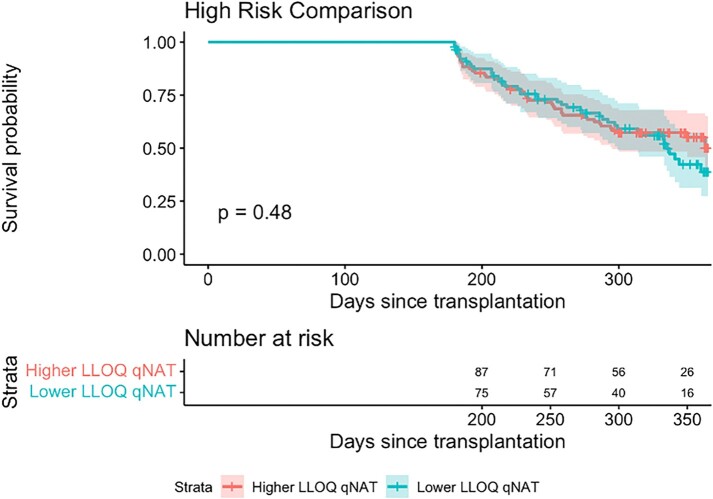

Kaplan Meier curve analysis comparing time to first episode of CMV DNAemia for higher LLOQ high risk patients (n=103) with lower LLOQ high risk patients (n=90) over a one year follow up period after transplantation. A log-rank test was performed and showed no significant difference (p-value = 0.48) in time to CMV DNAemia between the two groups. The median survival for the lower LLOQ group was 335 days and could not be calculated for the higher LLOQ group given that the survival probability was greater than 50% at the end of the study period.

**Conclusion:**

qNAT assays with a lower LLOQ result in earlier CMV DNAemia detection and longer antiviral treatment durations in moderate-risk RTRs without affecting all-cause mortality. These findings have important implications for healthcare associated costs and antiviral stewardship.

**Disclosures:**

**All Authors**: No reported disclosures

